# Forecasting mergers and acquisitions failure based on partial-sigmoid neural network and feature selection

**DOI:** 10.1371/journal.pone.0259575

**Published:** 2021-11-17

**Authors:** Wenbin Bi, Qiusheng Zhang

**Affiliations:** School of Economics and Management, Beijing Jiaotong University, Beijing, China; Vellore Institute of Technology: VIT University, INDIA

## Abstract

Traditional forecasting methods in mergers and acquisitions (M&A) data have two limitations that significantly reduce forecasting accuracy: (1) the imbalance of data, that is, the failure cases of M&A are far fewer than the successful cases (82%/18% of our sample), and (2) both the bidder and the target of the merger have numerous descriptive features, making it difficult to choose which ones to forecast. This study proposes a neural network using partial-sigmoid (i.e., partial-sigmoid neural network [PSNN]) as the activation function of the output layer and compares three feature selection methods, namely, chi-square (chi2) test, information gain and gradient boosting decision tree (GBDT). Experimental results prove that our PSNN (improved up to 0.37 precision, 0.49 recall, 0.41 G-Mean and 0.23 F1-measure) and feature selection (improved 1.83%-13.16% accuracy) method can effectively improve the adverse effects of the defects of the above two merger data on forecasting. Scholars who studied the forecast of merger failure have overlooked three important features: assets of the previous year, market value and capital expenditure. The chi2 test feature selection method is the best among the three feature selection methods.

## Introduction

Mergers and acquisitions (M&A) are an important strategy of corporate management and development [1], and its success is also very important for bidders and the target. However, accurately forecasting M&A outcomes remains a challenging task [2]. Traditional M&A forecasting often uses linear superposition methods, such as logit and probit. With the development of research, numerous studies have demonstrated that such methods cannot solve some of the significant problems in forecasting M&A [3, 4], including unbalanced data, type II errors and non-linearity [5]. On the contrary, non-linear machine learning (ML) methods, such as neural networks, can better solve these problems by virtue of their good fitting properties. In recent years, neural network and other non-linear ML methods have been gradually applied to M&A forecast research. Kangbok et al. used neural networks to forecast the failure of M&A [5]; Bruno and Maxwell combined neural networks with other forecasting methods to identify M&A targets [6]; and Zhang et al. used neural networks, support vector machine (SVM), decision trees and random forests to forecast the success of M&A [4]. However, two problems still need to be solved. First, no appropriate neural network method exists that can deal with the problem of data class imbalance, and second, which features are more suitable for classification tasks remain unclear.

There is no doubt that success in M&A occurs far more than failure. The data of Wang and Branch only had 10% failure samples while in our data, only 18% failed [7]. The unbalanced structure of the data makes the neural network more inclined to ‘sacrifice’ the information contained in the minority class when classifying, thereby causing the neural network to have very low accuracy in forecasting merger failure. (For example, if the training set consists of 99 positive classes and 1 negative class, then the neural network only needs a learning machine that will return all samples as positive classes to guarantee 99% accuracy. However, such a learning machine is obviously meaningless, because it will not forecast failure at all.) In previous studies, resampling [8–10] and cost-sensitive learning [5] were often used to address imbalance in the category. Scholars have also constantly proposed new methods in recent years [10, 11], and imbalanced data methods have applied in different domains, such as intrusion detection [12], stroke prevention [11], predicting battery life [13]. From the perspective of improving the neural network itself, the present article uses the partial-sigmoid function as the activation function of the output layer. Our experimental result shows that the proposed partial-sigmoid neural network (PSNN) model can significantly improve the forecasting accuracy of failed samples as well as the precision, recall, G-Mean and F1-measure. What is exciting is that our proposed model preserves the integrity of the data set compared to the existing data resampling methods and has lower computational complexity than cost-sensitive learning.

In M&A forecasting research, regardless of whether a study uses simple logit model or a complex SVM, Bayesian classifier, or a neural network, a problem that cannot be avoided is how to choose the most appropriate relevant features (descriptive variables, predictive factors, etc.). This limitation includes two points. One, in real-world tasks, we often encounter the problem called the ‘curse of dimensionality’, wherein the amount of calculation increases exponentially as the dimensionality increases. This is caused by the use of too many features. If we can select some of the most important features for our classification task and use these for model construction and training, then the curse of dimensionality can be greatly reduced. Two, when the classification machine is trained, it continuously extracts the classification information hidden in the features. Removing the useless features or adding related features may reduce the difficulty of the learning task and thereby improve the forecasting accuracy.

In previous M&A forecasting studies, Amir and Geoff used 12 sample characteristics and 8 deal characteristics to forecast the investment income of a bidder after M&A [14]. Moreover, on the basis of relevant literature, Kangbok et al. used 11 financial/accounting predictors and 12 M&A predictors to forecast the success or failure of a merger [5], while Bruno and Maxwell proposed eight hypotheses and a total of 35 variables to forecast the acquisition target [6]. However, whether these features (i.e. characteristics, predictors, variables) can provide effective information to the learning machine has not been determined. Therefore, in the current work, we innovatively use feature selection for data pre-processing. Scholars in the area of ML have proposed many feature selection methods to cope with the large number of features in research [10]. This article uses three common feature selection methods for ML, namely, chi-square (chi2) test, information gain and gradient boosting decision tree (GBDT), to select 16 features from 35 bidders’ features for comparison experiments. Our experimental results show that the feature selection program finds important features that are ignored by scholars and significantly improves the forecasting accuracy.

The contributions of this article can be found in several innovations presented here. (1) For M&A researchers, using feature selection for data pre-processing can alleviate the curse of dimensionality, increase the efficiency of non-linear ML and improve forecasting accuracy. Our improved PSNN model can also provide more ideas for future research. (2) For business managers, the features selected in the feature selection process often imply more important information. Focusing on these features will help managers make more accurate decisions. (3) In terms of information, investors are often lagging behind managers, which means that investors often have higher requirements for the accuracy of forecasts. Both feature selection and our improved PSNN can significantly improve the forecasting accuracy.

The remainder of this paper is organized into sections. Section 2 describes the two basic methods of unbalanced data and the improved PSNN method proposed in this study. Section 3 then introduces the three feature selection methods we used. Section 4 summarises the data used in this article. Section 5 presents the analysis of experimental results, and Section 6 presents the conclusions.

## Class imbalance data and PSNN

In this section, we will discuss two basic methods of dealing with class imbalances and the PSNN method.

### Data-oriented approach

The under-sampling method involves randomly removing some samples from the majority samples so that the number of positive and negative classes in the processed sample set is close to each other, followed by training the model with a more balanced data set. Random under-sampling is a typical under-sampling method. For example, the training sample *E* consists of the majority class sample set *E*_*maj*_ and the minority class sample set *E*_*min*_ (*E*_*maj*_∪*E*_*min*_ = *E*, *E*_*maj*_∩*E*_*min*_ = ∅). The eliminated sample set *E*_0_ (*E*_0_⊂*E*_*maj*_) is selected from *E*_*maj*_, and *E*−*E*_0_ is used as the training set of the neural network. When the random under-sampling method removes majority class samples to form balanced data, it destroys the integrity of the training set. However, the removed samples may contain important information required by the classification machine, thus resulting in missing information. To resolve this problem, Liu proposed the EasyEnsemble and BalanceCascade algorithms to overcome the deficiency [15]. The ensemble of under-sampling divides the majority category into several subsets and combines these subsets with the minority class to avoid missing information [16].

The over-sampling method expands the number of samples of the minority class so that the numbers of positive and negative classes are close to each other. The basic idea of random over-sampling is to analyse the samples of minority classes and add new samples that are copied from the minority class samples to the data set. For random over-sampling, because part of the minority samples is copied, on the one hand, the training data set becomes bigger and the training process necessarily takes much longer. On the other hand, if there are some noise points in the sample, these noise points may be doubled when these samples are copied. Besides, it is obvious that over-sampling can easily lead to overfitting. The iconic algorithms for oversampling are the SMOTE algorithm [17], Borderline-SMOTE algorithm [18], ADASYN [19] and Boundary-Boost [20].

The over-sampling method shows better performance in processing class overlapping samples [21]. In comparison, random under-sampling is generally more effective when there is noise in the data set [22]. In recent years, scholars have continued to optimize the sampling method [23–28]. Even though the sampling methods have continued to optimise the process of determining class imbalance, they also destroy the original structure of the data set.

### Cost-sensitive learning

Resampling technology solves the problem of data imbalance at the data level, whereas cost-sensitive learning solves the problem of data imbalance at the algorithm level. Most classification algorithms assume that there is no significant difference in the number of various types of samples. However, in the classification problem with unbalanced data, the cost of classifying the majority class into the minority class is far less expensive than that of classifying the minority class into the majority class [5]. Therefore, cost-sensitive learning applies the cost matrix (1) to weigh the classification results:

$$cost=[\begin{array}{cc} {cost}_{00} & {cost}_{01}\\ {cost}_{10} & {cost}_{11} \end{array}],$$ (1)

where *cost*_*ij*_ represents the cost of classifying the i-th category into the j-th category, and generally, *cost*_*ii*_ = 0.

The realisation of cost-sensitive learning methods can be embodied in the pre-processing of the training data and the post-processing of the output and direct cost-sensitive learning. Cost-sensitive algorithms have been widely combined with various classification methods, such as SVM [29, 30], decision trees [31–33] and neural networks [34]. Cost-sensitive learning often requires more training time due to the additional calculations required in the training process.

### Backpropagation neural network (BPNN) with optimised output layer activation function (PSNN)

The iterative goal of the backpropagation (BP) algorithm of the neural network is to minimise the cumulative error on the training set *E*

$$e=\frac{1}{m}\sum_{k=1}^{m}e_{k},$$ (2)

where *m* is the sample size of the training set, and *e*_*k*_ is the mean square error of the kth sample (*x*_*k*_, *y*_*k*_). In addition,

$$e_{k}=\frac{1}{2}{\left(y_{k}-{\hat{y}}_{k}\right)}^{2},$$ (3)

where *y*_*k*_∈{0,1), and ${\hat{y}}_{k}$ is the output of the neural network after this iteration.

**Fig 1:**
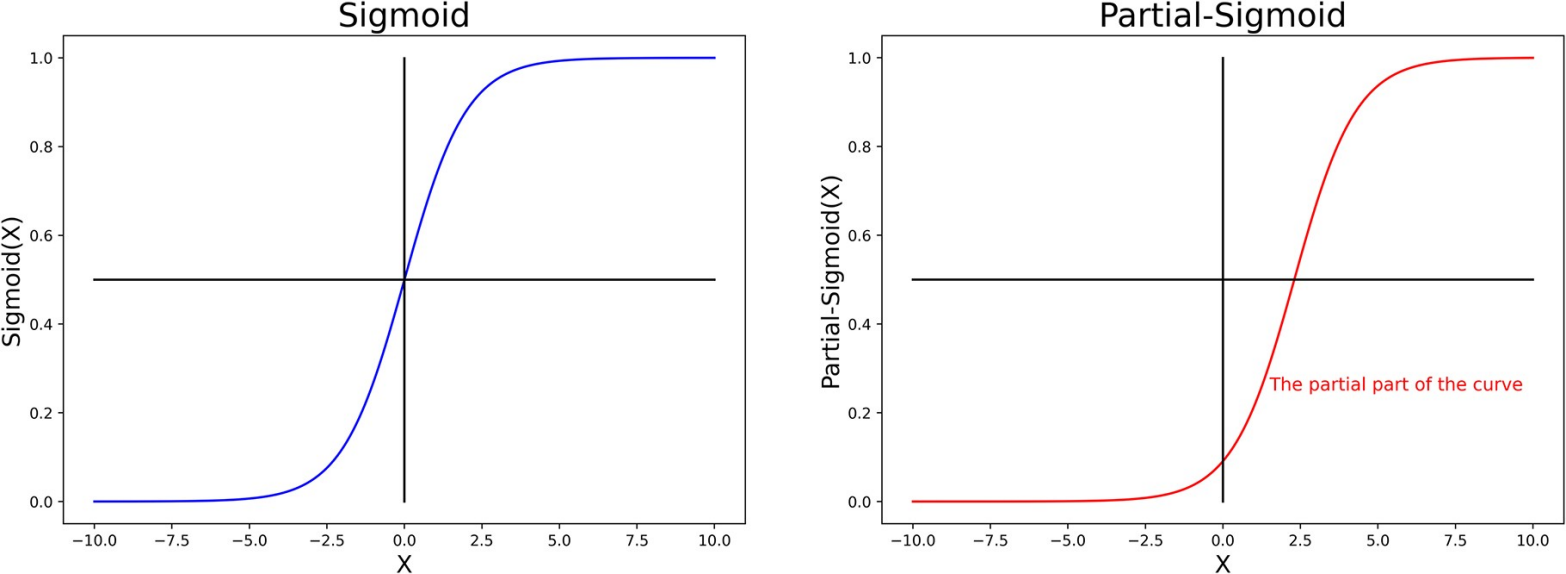
Sigmoid function and partial-sigmoid function.

Neural network models for binary classification problems, such as merger forecasting, often use the sigmoid function (or tanh function, with categories of 1 and −1) as the activation function of the output layer. However, the sigmoid function is not sensitive to unbalanced data sets, that is, the result output by the hidden layer is ‘equally distributed’ by the sigmoid function to the positive class (merger success) and the inverse class (merger failure). Therefore, the partial-sigmoid function is used as the activation function of the output layer (Fig 1). We call this the PSNN.


$$sigmoid(x)=\frac{1}{1+e^{-x}}$$ (4)

$$partial-sigmoid(x)=\frac{1}{1+{ne}^{-x}}$$ (5)

**Fig 2:**
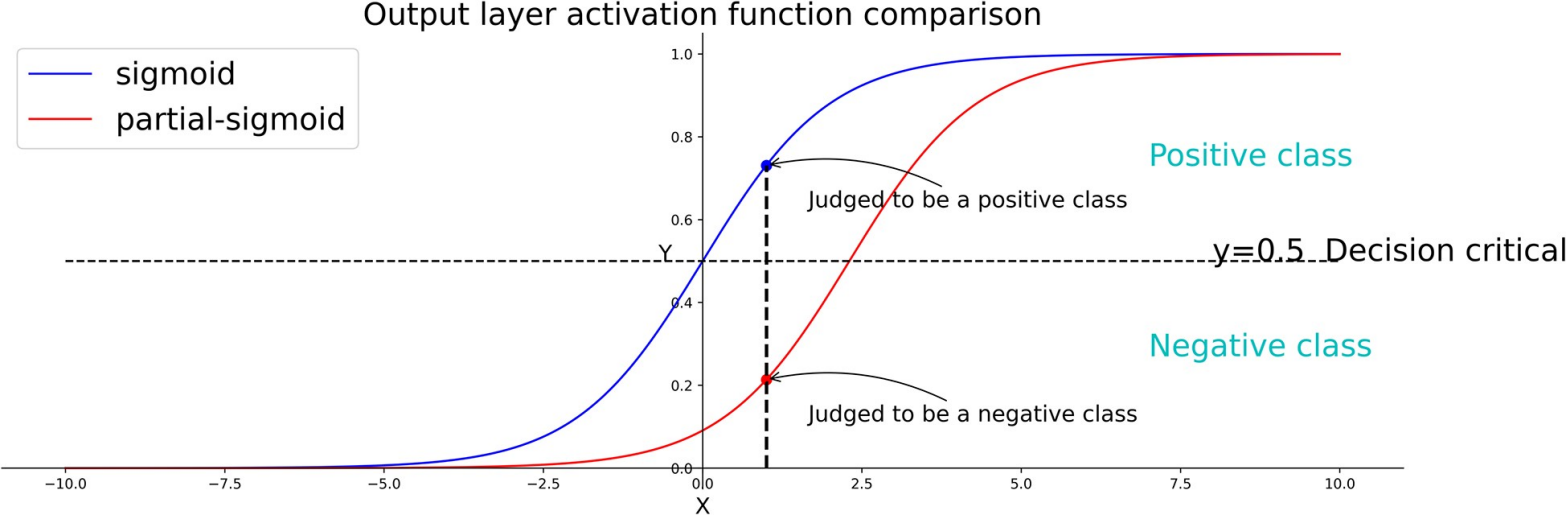
Decision comparison between the sigmoid and partial-sigmoid functions.

Using partial sigmoid as the output layer activation function has several advantages, which are listed below:

1. The partial-sigmoid function is biased to 0, which makes it easier for the neural network to output 0 (i.e. minority). It is also more sensitive to the minority class, thus encouraging the model to classify the sample into the minority class (Fig 2). The partial-sigmoid function is similar to cost-sensitive learning in that the penalty for misclassification of the minority class into the majority class is greater.

2. The formula for the error BP is *ω*_*i*_←*ω*_*i*_+Δ*ω*_*i*_. The error BP is based on the gradient descent method ($\Delta \omega_{i}=-\eta \frac{\partial E}{\partial \omega_{i}}$, where *η* is the learning rate, and $\frac{\partial E}{\partial \omega_{i}}=\frac{\partial E}{\partial \hat{y}}∙\frac{\partial \hat{y}}{\partial \omega_{i}}$).

Compared with the BPNN, when PSNN and BPNN are initialised with the same initial input layer and hidden layer weight, the partial sigmoid will get a larger initial cumulative error. This indicates that the gradient of our proposed model will descend faster and require fewer computations to iterate to the optimum.

3. The sigmoid function has a good property, that is, *f*′(*x*) = *f*(*x*)(1−*f*(*x*)). The partial-sigmoid function perfectly maintains this property: ${\left(\frac{1}{1+{ne}^{-x}}\right)}^{\prime }=\frac{{ne}^{-x}}{{\left(1+{ne}^{-x}\right)}^{2}}=\left(\frac{1}{1+{ne}^{-x}}\right)\left(\frac{{ne}^{-x}}{1+{ne}^{-x}}\right)=\left(\frac{1}{1+{ne}^{-x}}\right)\left(1-\frac{1}{1+{ne}^{-x}}\right)$. Therefore, it will not have any adverse effect on the neural network model itself.

## Feature selection

In the development of ML feature selection, many feature selection methods have been produced, such as PCA [35, 36], KPCA [15, 37], LDA [38, 39], GA [40] and simulated annealing [41]. Feature selection has many classification methods. Feature selection is also divided into supervised, unsupervised and semi-supervised based on the supervision method. According to the search strategy, feature selection is divided into global, random and heuristic search strategy. In addition, feature selection can be divided into algorithms, such as distance, consistency, dependency and information measurement, based on the evaluation criteria used. Finally, according to the combination of feature selection and ML methods, feature selection can be divided into filter, wrapper and embedded. This section uses the three feature selection methods (chi2 test, information gain, GBDT).

### Chi2 test

The Chi2 test proposed by Pearson is used to measure the relevance of features to labels. It belongs to the category of non-parametric tests and mainly compares two or more sample rates (composition ratios). It also carries out a correlation analysis of two categorical variables. Chi2 test is widely used in feature selection [42–44] and is calculated as follows:

$$\chi^{2}=\sum \frac{{\left(O-E\right)}^{2}}{E},$$ (6)

where *O* is the observed frequency, and *E* is the desired frequency.

The degree of deviation between the actual observation value and the theoretical inferred value determines the size of the chi2 value. The larger the chi2 value, the greater the deviation between the aforementioned values; on the contrary, the smaller the chi2 value, the smaller the deviation. If the actual observation value and the theoretical inferred value are completely equal, then the chi2 value becomes 0, indicating that the theoretical value is completely in line with reality.

### Information gain

Information gain is an effective feature selection method. In forecasting the probability distribution of a random event, our forecast should meet all known conditions, and we should not make any subjective assumptions about the unknown. In this case, the probability distribution is the most uniform and the forecasted risk is the smallest. Szidónia and László [45] combined information gain with Gabor filter for feature selection. Azhagusundari and Antony combined information gain with the discernibility matrix [46]. Information gain is also used in various aspects, such as text classification [47, 48] and credit risk [43].

For a data set E, the probability of class *i* samples is *P*_*i*_(i = 0,1, for two classification problems). For a certain feature A, the data set E is divided into V subsets according to its value. Thus, the information gain of A is expressed as

$$gian(A)=Ent(E)-\sum_{k\in V}\frac{\left|E_{k}\right|}{\left|E\right|}Ent(E_{k}).$$ (7)

Note that A is assumed to be a discrete variable. If A is a continuous variable, then Formula (7) will need to be changed slightly.

*Ent*() represents information entropy, and its calculation formula is given by

$$Ent(E)=-(p_{0}{log}_{2}p_{0}+p_{1}{log}_{2}p_{1}).$$ (8)

### GBDT

Decision tree is a traditional ML method. It uses a root-to-leaf construction method to generate a tree, after which its selects features and determines feature values at the branch nodes of the decision tree. Then, it branches down from the branch node according to the optimal feature value. The leaf nodes of the decision tree get the classification results. Therefore, each path from the root node to the leaf node of the tree corresponds to a branching rule. The entire decision tree corresponds to a set of classification expression rules, which are the combinations of features (i.e. the feature selection capability of the tree model). However, the classification ability of a single tree is weak; thus, multiple trees can be combined to make a joint decision and response. The most famous ones are the random forest method based on bagging ideas and the GBDT method based on boosting ideas. Different from the parallel and relatively independent forms of trees in the random forest, the trees in GBDT are generated in series, and each tree is generated in the direction of reducing the residual error of the previous tree. Thus, the iteration speed of the spanning tree is faster, and the establishment of an integrated tree is more efficient.

GBDT is a feature selection method based on decision tree [49]. Function approximation is a numerical optimisation from the aspect of function space, which combines stage-wise additive expansions and steepest-descent minimisation. In addition to being used for feature selection [50], GBDT is also used for regression [51] and classification [52]. Unlike the traditional decision tree method, which weighs positive and negative samples, GBDT makes the algorithm converge globally by following the direction of negative gradient [53]. During the generation of each tree, the residual of the previous tree is calculated, after which the fitting is carried out on the basis of the residual. In the process of continuously generating the tree, the residual is continuously reduced, and the fitted value gradually gets closer to the actual value.

## Data

The M&A transaction data used in this study came from the iFinD database. A total of 37,997 transaction sample data from January 1, 2015 to December 31, 2019 were obtained, and the financial data and financial indicator data of listed companies were obtained from the CSMAR and JQData databases. All financial data and financial indicator data were selected from the date of the first announcement of the M&A transaction. If there were no corresponding data on the date of the first announcement, the latest data before the first announcement were selected. Next, the data were processed as follows:

1. Removal of the non-equity transactions.
2. Removal of transactions for which business control has not been transferred, as M&A is a transaction based on company control [54].
3. Removal of unfinished M&A.
4. Removal of the increase or decrease of stock holdings as well as the gifting and transfer of stocks that do not constitute an M&A transaction.
5. Removal of transactions with incomplete financial data and financial indicator data in CSMAR and JQData.

After the above sample selection process, we finally obtained 874 M&A transactions. Among them, 717 (~82.0%) were successful samples and 157 (~18.0%) were failed samples. This ratio proves that the success and failure of M&A are not balanced. Among the 874 M&A transactions, 774 M&A transactions were randomly selected as the training set and the remaining 100 M&A transactions were used as the test set. The training set had 636 (82.2%) successful samples and 138 (17.8%) failed samples. The test set had 81 (81.0%) successful samples and 19 (19.0%) failed samples. The following will explain why the data set was divided in this way.

In terms of features, we collected 35 feature data about bidders, three feature data about target parties and two feature data about M&A transactions. The 35 feature variables about bidders came from two sources: the feature variables contained in the listed company’s own financial announcements and the conclusions from relevant references [5, 6]. The detailed feature variables and descriptive statistics of our data are shown in Tables 1 and 2, respectively.

**Table 1:** Feature variables.

Serial number	Feature variables	Description
**Original features of bidders**		
V01	Inventory	We obtained the financial data on or closest to the first announcement date of M&A.
V02	Total assets	
V03	Assets of last year	
V04	Assets three years ago	
V05	Market value	
V06	Shareholders’ equity	
V07	Total dividend	
V08	Working capital	
V09	Operating income	
V10	Capital expenditure	
V11	Net sales	
V12	Operating revenue	
V13	Net profit	
V14	EBIT this year	
V15	EBIT last year	
V16	EBIT three years ago	
**Structural features of bidders**		
*Inefficient management*		
V17	ROA	
V18	ROE	
V19	Inventory/Total assets	
V20	EBIT/Operating revenue	
V21	DPS	Dividend per share
V22	Asset turnover	Net sales/Total assets
V23	Net profit/Market value	
V24	Inventory/Working capital	
*Undervaluation*		
V25	M/b ratio	Market value of assets/Book value of assets
V26	P/E ratio	Closing price/Earnings per share
*Growth-resource*		
V27	Growth in sales over the past year	(This year − t years ago)/t years ago
V28	Growth in EBIT over the past year	
V29	Growth in EBIT over the past three years	
V30	Growth in total assets over the past year	
V31	Growth in total assets over the past three years	
V32	Capital expenditure/Operating revenue	
*Dividend payout*		
V33	Dividend/Shareholders’ equity	
V34	Dividend payout ratio	Total dividends/Income before extraordinary items
*Size*		
V35	Log (total assets)	The natural log of total assets
**Features of targets**		
V36	Total value	
V37	Book value of assets	
V38	Asset appraisal value	
**Features of M&A**		
V39	Bidder paid cash	
V40	All-cash deal	

**Table 2:** Descriptive statistics.

Serial number	Feature variables	mean	std	Serial number	Feature variables	mean	std
V01	Inventory	2.27E+09	1.53E+10	V21	DPS	0.14	1.04
V02	Total assets	1.01E+10	2.99E+10	V22	Asset turnover	1.03	1.09
V03	Assets of last year	8.28E+09	2.35E+10	V23	Net profit/Market value	0.01	0.01
V04	Assets three years ago	5.66E+09	1.77E+10	V24	Inventory/Working capital	4.29	98.23
V05	Market value	1.05E+10	1.65E+10	V25	M/b ratio	3.02	4.91
V06	Shareholders’ equity	4.24E+09	9.29E+09	V26	P/E ratio	9.07	1331.84
V07	Total dividend	1.05E+08	4.29E+08	V27	Growth in sales over the past year	40.12	239.75
V08	Working capital	1.37E+09	8.64E+09	V28	Growth in EBIT over the past year	0.77	14.00
V09	Operating income	1.32E+09	2.91E+09	V29	Growth in EBIT over the past three years	-62.56	1915.22
V10	Capital expenditure	3.33E+08	9.78E+08	V30	Growth in total assets over the past year	0.33	1.16
V11	Net sales	8.97E+09	2.10E+10	V31	Growth in total assets over the past three years	2.52	33.32
V12	Operating revenue	5.00E+09	1.15E+10	V32	Capital expenditure/Operating revenue	0.11	0.20
V13	Net profit	9.15E+07	2.97E+08	V33	Dividend/Shareholders’ equity	0.02	0.02
V14	EBIT this year	1.16E+08	3.77E+08	V34	Dividend payout ratio	2.25	25.84
V15	EBIT last year	1.04E+08	4.19E+08	V35	Log (total assets)	22.01	1.27
V16	EBIT three years ago	5.80E+07	3.49E+08	V36	Total value	1.13E+05	3.54E+05
V17	ROA	1.21	4.14	V37	Book value of assets	4.89E+04	3.12E+05
V18	ROE	-101.21	3046.82	V38	Asset appraisal value	1.14E+05	3.56E+05
V19	Inventory/Total assets	0.14	0.13	V39	Bidder paid cash	4.54E+04	1.40E+05
V20	EBIT/Operating revenue	0.21	2.40	V40	All-cash deal	0.54	0.50

## Experimental results

All feature selection and classification techniques in this article are executed in a Python 3.7 environment. All the computations were performed on a computer system with MacOS 10 operating system, 8 GB 2133 MHz LPDDR3 and Intel Core i5 processor. Libraries of Python, such as Pandas and NumPy, were used to test the classification models, while libraries, such as sklearn, were used for the feature selections.

### Feature selection for balanced data sets

The above three commonly used feature selection methods in ML (chi2 test, information gain and GBDT) were compared to confirm whether feature selection can improve classification accuracy. We under-sampled the data set because whether or not it is balanced would not affect our conclusions. In this experiment, we used a BPNN with 1 hidden layer and 5 neurons. Data pre-processing was carried out as follows:

S1: Perform random under-sampling by forming a sample size of 314 balanced data (157 successes, 157 failures). This is followed by random sampling to form a training set with 276 data (137 successes, 139 failures) and a test set with 38 data (20 successes, 18 failures).

S2: Perform feature selection on 35 bidder features to form five comparative experimental groups (G1: 35 features of all bidders, G2: 19 structural features of bidders, G3: 16 features of bidders selected by the chi2, G4: 16 features of bidders selected by the information gain, G5: 16 features of bidders selected by the GBDT).

S3: Add M&A and target features and convert symbolic features into dummy variables.

S4: Conduct data standardisation. Given that the dimension difference between different features is very large (the mean value in descriptive statistics can prove this), the entire data space is ‘pulled’ very long in some dimensions, and some dimensions are ‘compressed’ to be very short. This makes the importance of ‘stretched’ features in classification much greater than that of ‘compressed’ features, which is not conducive to the neural network in finding the dividing line in the data space. Standardisation limits the data space to the same order of magnitude without changing the relative position of each group of data. Here, we use the following formula to standardise:

$$Normalised-X=\frac{X-\mu }{\sigma }.$$ (9)

S5: Training and testing.

The experimental results obtained are shown in Table 3.

**Table 3:** Feature selection results.

Information gain		Chi2		GBDT	
**V03**	**Assets of last year**	V01	Inventory	V01	Inventory
**V05**	**Market value**	V02	Total assets	**V03**	**Assets of last year**
V06	Shareholders’ equity	**V03**	**Assets of last year**	**V05**	**Market value**
V07	Total dividend	V04	Assets three years ago	**V10**	**Capital expenditure**
V08	Working capital	**V05**	**Market value**	V17	ROA
V10	Capital expenditure	V06	Shareholders’ equity	V18	ROE
V12	Operating revenue	V07	Total dividend	V19	Inventory/Total assets
V13	Net profit	V09	Operating income	V20	EBIT/Operating revenue
V15	EBIT last year	**V10**	**Capital expenditure**	V22	Asset turnover
V22	Asset turnover	V11	Net sales	V23	Net profit/Market value
V23	Net profit/Market value	V12	Operating revenue	V26	P/E ratio
V25	M/b ratio	V13	Net profit	V27	Growth in sales over the past year
V26	P/E ratio	V14	EBIT this year	V28	Growth in EBIT over the past year
V29	Growth in EBIT over the past three years	V25	M/b ratio	V30	Growth in total assets over the past year
**V33**	**Dividend/Shareholders’ equity**	**V33**	**Dividend/Shareholders’ equity**	**V33**	**Dividend/Shareholders’ equity**
V34	Dividend payout ratio	V35	Log (total assets)	V34	Dividend payout ratio

The three feature selection methods all consider that V03 (Assets of last year), V05 (Market value), V10 (Capital expenditure) and V33 (Dividend/shareholders’ equity) are more favourable features for classification. On the contrary, V03, V05 and V10 are ignored by previous scholars. This finding proves the importance of adding feature selection in M&A forecasting research so that it can find those hidden features that are most suitable for forecasting, regardless of whether there is previous literature that proves these features are beneficial for classification or forecasting.

Table 4 shows the forecasting results of five sets of samples when iterating 2000 times. By comparing group 2 and groups 3, 4 and 5, we can clearly find that forecasting accuracy is significantly improved and errors are reduced after feature selection. This outcome further confirms that researchers ignored some very important features in previous M&A forecasting efforts. By identifying these ignored important features, the feature selection proposed in this study greatly improves the classification accuracy. The results also show that the proposed feature selection can effectively alleviate the dimensionality disaster. In particular, the iteration time is reduced by nearly half after feature selection. This is because group 1 needs to calculate 205 parameters whilst in groups 3, 4 and 5, only 110 parameters need to be calculated. The chi2 test also performs better than other methods in both training data and test data.

**Table 4:** Results of training 2000 times.

Iteration: 2000 Learning rate: 0.02	G1: All features		G2: Structural features		G3: Chi2		G4: Information gain		G5: GBDT	
Number of features	35+5		19+5		16+5		16+5		16+5	
	Training data	Test data	Training data	Test data	Training data	Test data	Training data	Test data	Training data	Test data
Sample size	276	38	276	38	276	38	276	38	276	38
Correct classification	193	21	185	17	199	21	195	19	190	18
Accuracy	69.93%	55.26%	67.03%	44.74%	72.10%	55.26%	70.65%	50.00%	68.84%	47.37%
Iteration error	27.77	—	28.83	—	26.9	—	27.29	—	27.99	—
Iteration time(s)	508.85	—	315.71	—	292.83	—	289.42	—	297.06	—

Table 5 shows the forecasting results when the neural network is iterated to the lowest error. In terms of accuracy, similar to the above table, the best performance is shown by chi2 test, in which accuracy of the training data and the test data are 80% and 60%, respectively. Although the performances of information gain and GBDT are slightly worse than that of the non-feature selection group, they are also completely better than the performance of the scholars’ structural features. After feature selection, the accuracy is improved by up to 12%. Furthermore, the errors in groups 1 and 3 are close, but the accuracy differs by 1.5% and 5.27%, showing that irrelevant features have an adverse effect on the classifier.

**Table 5:** Training to optimal results.

Learning rate: 0.02	G1: All features		G2: Structural features		G3: Chi2		G4: Information gain		G5: GBDT	
Number of features	35+5		19+5		16+5		16+5		16+5	
	Training data	Test data	Training data	Test data	Training data	Test data	Training data	Test data	Training data	Test data
Sample size	276	38	276	38	276	38	276	38	276	38
Correct classification	217	21	199	18	221	23	210	19	204	20
Accuracy	78.62%	55.26%	72.10%	47.37%	80.07%	60.53%	76.09%	50.00%	73.91%	52.63%
Iteration error	19.36	—	22.85	—	19.57	—	21.58	—	20.79	—

In summary, the experimental results in this section enable us to confirm two points. First, compared with the structural sample feature combination, the sample feature combination produced by feature selection can significantly improve forecasting accuracy. Second, feature selection can effectively alleviate the dimensional disaster and reduce the iteration time by nearly half.

### Data splitting

Data splitting divides data into two parts. One part is used for model training (training set) and the other part is used for model verification (test set). Reasonable data splitting can help obtain a more reliable forecasting distribution model [55]. In case the amount of data is sufficient, the data should be divided into 64% training set and 36% test set [56]. However, because financial data, such as M&A, are different from other classification tasks, such as text classification and image recognition, we cannot obtain enough effective data for training and recognition. Therefore, to obtain more data for training, the data are divided into 89% training set and 11% test set.

**Fig 3:**
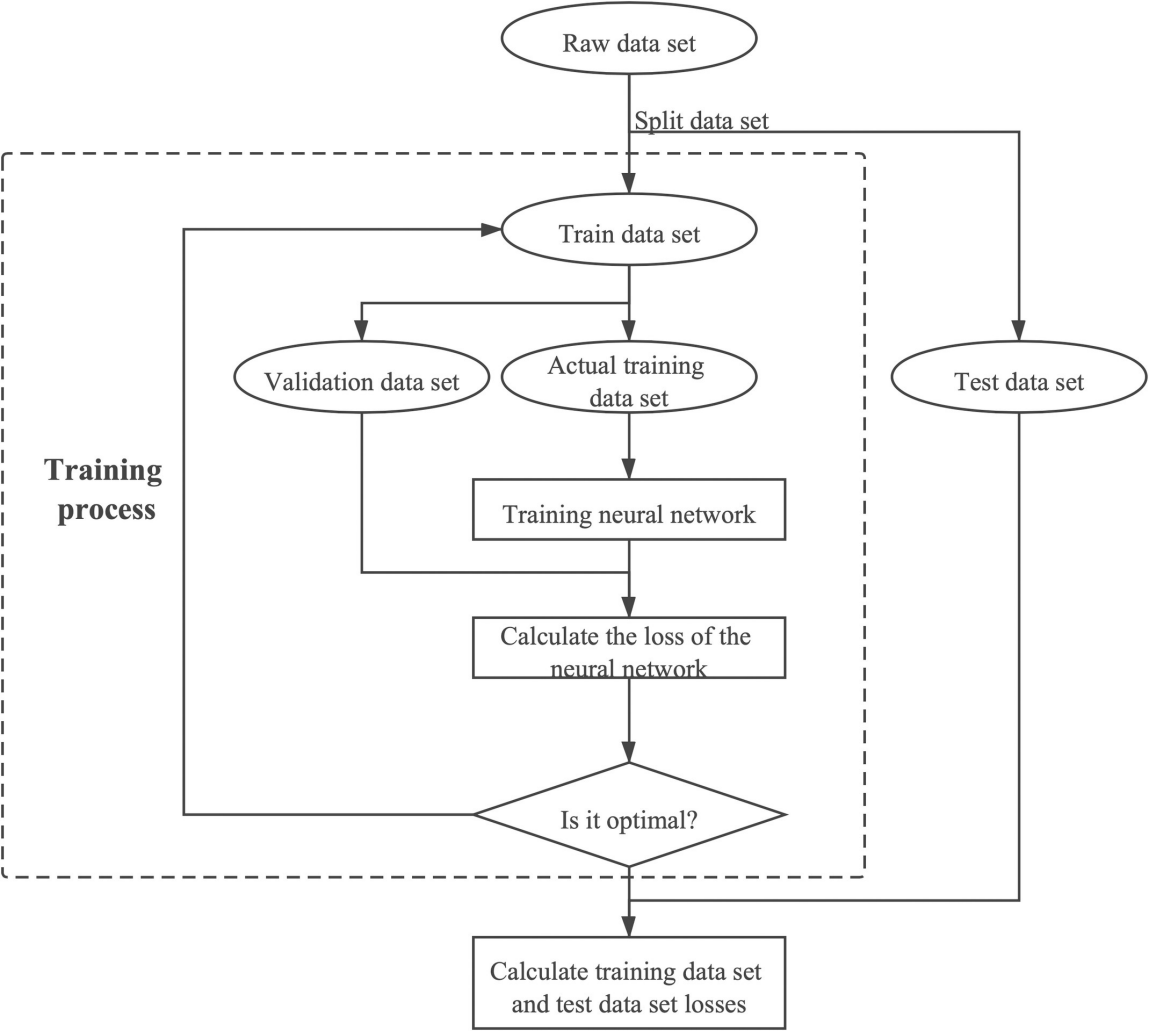
Training process.

In addition, we use cross-validation to train the neural network [57], because neural network training is prone to overfitting (i.e. the accuracy of the neural network in the training set is very high, but the accuracy in the test set is very low). In this method, in addition to dividing the data into training set and test set, we also need to split the training set into sub-training set and validation set. Specifically, the original data set is divided into three parts: training set, validation set and test set. Every part is involved in the training of the training set only. In addition to training after each inspection error, the validation set error is also tested outside the training set, The purpose of training is to minimise the overall error on the training set and validation set. The data of the test set will not be used for model construction but only for the final testing of the model’s accuracy. This method guarantees the best model without overfitting. In our experiment, each iteration draws 10% of the training set as the validation set. When the next iteration is completed, the validation set is emptied and re-drawn to ensure that all training set samples are used for training. Fig 3 shows training process in our experiment.

### Evaluation index for unbalanced data

In traditional classification research, classification accuracy is often used as an evaluation criterion to measure the effectiveness of algorithms. However, classification accuracy cannot reflect the classification accuracy of minority classes [58]. The form of the confusion matrix is generally adopted in imbalance classification [59]. Table 6 shows the confusion matrix commonly used to measure the effectiveness of algorithms in unbalanced data tasks.

**Table 6:** Confusion matrix.

	Positive forecast	Negative forecast
Positive class	TP	FN
Negative class	FP	TN

The focus of this article is to increase the sensitivity of neural networks to merger failures. Therefore, in this experiment, positive represents merger failure, and negative represents merger success. This article will use the following four evaluation indicators:

$$precision=\frac{TP}{TP+FP},$$ (10)

$$Recall=\frac{TP}{TP+FN},$$ (11)

$$G-Mean=\sqrt[{2}]{\frac{TP}{TP+FN}\times \frac{TN}{TN+FP}},$$ (12)

$$F_{1}-measure=\frac{Accuracy\times Recall}{Accuracy+Recall},$$ (13)

where *precision* is the proportion of true M&A failures among all judged M&A failures, *Recall* suggests that the proportion of M&A fails to correctly judge the actual accounting for M&A failure, *G*−*Mean* examines the classification accuracy of the majority class and the minority class at the same time (only when the classification accuracy of the majority class and the minority class are higher at the same time can a higher *G*−*Mean* be obtained) and *F*_1_−*measure* is the harmonic mean of *precision* and *Recall*.

### Experimental results with unbalanced data

The BPNN is used in this research. In theory, when there are enough neurons, a single hidden layer neural network can approximate any non-linear function. Unfortunately, there is currently no relevant research to guide us on how to determine the number of neurons. Generally, we need to keep the number of neurons from getting too large so that we would not overfit the neural network in the training data set, thus resulting in poor forecasting in the test data set, which is not the result we want. We consulted some literature and carried out some tests. Finally, we chose a BPNN with three neurons to conduct our experiments. To make our conclusions more convincing, a supplementary experiment was likewise conducted with 5 and 10 neurons. The feature selection method in this experiment is the chi2 test, which performed best, as shown in Section 5.1.

Table 7 shows the logit model, the BPNN model with the standard sigmoid activation function and the proposed PSNN model with the output layer activation function, where the parameter n is the coefficient of *e*^−*x*^ in (5). Obviously, when n = 1, it is a standard neural network model.

**Table 7:** Comparative experiment of unbalanced data.

	Logit		PSNN		PSNN		PSNN		PSNN		PSNN	
			n = 1 (General BPNN)		n = 2		n = 3		n = 5		n = 10	
***3 hidden-layer neurons***	train	test	train	test	train	test	train	test	train	test	train	test
**precision**	0.10	0.09	0.23	0.18	0.36	0.31	0.38	0.32	0.38	0.33	0.29	0.28
**recall**	0.15	0.16	0.22	0.21	0.46	0.53	0.53	0.58	0.62	0.74	0.69	0.84
**G-Mean**	0.33	0.31	0.43	0.40	0.62	0.62	0.65	0.64	0.70	0.69	0.66	0.64
**F1-measure**	0.06	0.06	0.11	0.10	0.20	0.20	0.22	0.21	0.24	0.23	0.20	0.21
***5 hidden-layer neurons***												
**precision**	0.10	0.09	0.25	0.16	0.39	0.27	0.41	0.31	0.41	0.30	0.32	0.28
**recall**	0.15	0.16	0.23	0.21	0.49	0.47	0.55	0.58	0.64	0.68	0.70	0.89
**G-Mean**	0.33	0.31	0.44	0.39	0.64	0.58	0.67	0.64	0.72	0.66	0.69	0.64
**F1-measure**	0.06	0.06	0.12	0.09	0.22	0.17	0.23	0.20	0.25	0.21	0.22	0.21
***10 hidden-layer neurons***												
**precision**	0.10	0.09	0.31	0.11	0.44	0.26	0.47	0.29	0.47	0.29	0.36	0.26
**recall**	0.15	0.16	0.27	0.11	0.50	0.58	0.57	0.63	0.64	0.79	0.72	0.89
**G-Mean**	0.33	0.31	0.48	0.29	0.66	0.59	0.70	0.64	0.74	0.65	0.72	0.59
**F1-measure**	0.06	0.06	0.14	0.05	0.23	0.18	0.26	0.20	0.27	0.21	0.24	0.20

Three conclusions can be drawn from the summary of model performance displayed in Table 7. First, similar to the conclusions of many scholars, the neural network model performs better than the logit model in all aspects. Second, the four proposed PSNN models significantly improve the accuracy of classification in both the training data and the test data. Third, when n increases, the accuracy of classification is observed to have an abnormal phenomenon in the upward trend. When n increases from 5 to 10, each index drops abnormally. By observing the classification results of each sample more carefully, we find that the forecasting accuracy of the majority class is greatly reduced when the value of n gets too large.

Two supplementary comparative experiments revealed that the proposed PSNN (n = 5) model of 10 hidden-layer neurons does not perform better than all other models in terms of test data forecasting, despite it being the best performing model for training data forecasts. In other words, a higher training data fit does not lead to better test data forecasting.

## Conclusion and future work

### Conclusion

The imbalance of the class of M&A transactions and the selection of related characteristics are two major problems that plague the research on M&A failure forecasting. This study proposed a PSNN model to cope with the problem of class imbalance and applied feature selection methods to find the most suitable features for classification forecast. Through feature selection, we found three important classification features that were ignored by previous scholars: assets of last year, market value and capital expenditure.

Five years’ worth of M&A data were used for our experiments. Experimental results show that the features selected by the feature selection method can significantly improve the accuracy of classification and reduce the dimensionality disaster caused by a large number of features. The chi2 test showed the best performance among the three feature selection methods,. In addition, the comparative experiment on unbalanced data show that our improvement has breakthrough gains in forecasting unbalanced data by the neural network. More broadly, PSNN and feature selection can not only be used to forecast M&A failure but also to identify merger targets and assist in M&A decision making. Furthermore, the feature selection method be used for any merger forecasting, whether it is classification or regression.

### Limitations and future work

As reflected in the article, there are still some problems that need to be solved in the research process:

1. The number of features. Although feature selection can select the most favourable features for forecasting, it remains uncertain what the most appropriate number of features is. Too many features would lead to slow iteration and too long program running time. Conversely, too few features would inevitably affect forecasting accuracy.
2. The number of neurons in the hidden layer. We also briefly described this in Section 5.4. The number of neurons dictates the complexity of the neural network and is closely related to the number of parameters we need to calculate. Thus, increasing the number of neurons would result in a neural network with stronger generalisation ability, but it would also mean a longer iteration time and the risk of overfitting easily.
3. The value of n. As shown in the experimental results, a larger value of n does not guarantee a better effect. What value of n can be used to obtain the most accurate forecasting model has yet to be identified. Furthermore, whether the value of n is related to the proportion of the majority class and the minority class is another unresolved issue.

We will do more research on these problems in the future.
